# Expression profiling of metalloproteinases and their inhibitors in synovium and cartilage

**DOI:** 10.1186/ar2013

**Published:** 2006-07-19

**Authors:** Rose K Davidson, Jasmine G Waters, Lara Kevorkian, Clare Darrah, Adele Cooper, Simon T Donell, Ian M Clark

**Affiliations:** 1 School of Biological Sciences, University of East Anglia, Norwich NR4 7TJ, UK; 2 Institute of Orthopaedics, Norfolk and Norwich University Hospital, Norwich NR4 7UY, UK

## Abstract

Cartilage destruction in osteoarthritis (OA) is thought to be mediated by two main enzyme families; the matrix metalloproteinases (MMPs) are responsible for cartilage collagen breakdown, whereas enzymes from the 'a disintegrin and metalloproteinase domain with thrombospondin motifs' (ADAMTS) family mediate cartilage aggrecan loss. Tissue inhibitors of metalloproteinases (TIMPs) regulate the activity of these enzymes. Although cartilage destruction in OA might be driven by the chondrocyte, low-grade synovitis is reported in patients with all grades of this disease.

Our earlier work profiling these gene families in cartilage identified a number of genes that are regulated in OA, which are hence implicated in the disease process. Because the synovium might contribute to cartilage-matrix destruction in OA, we have extended the screening in the current study. We have profiled *MMP*, *ADAMTS *and *TIMP *genes in both cartilage and synovium from patients with either OA of the hip or a fracture to the neck of femur (NOF), giving a more complete picture of proteolysis in this disease.

The four most significantly upregulated genes (*P *< 0.0001) in OA synovium compared to the fractured NOF are *MMP28*, *ADAMTS16*, *ADAMTS17 *and *TIMP2*. For *MMP9*, *MMP10*, *MMP12*, *MMP17, MMP23*, *MMP28*, *ADAMTS4*, and *ADAMTS9*, there is a significant correlation between expression levels in the synovium and cartilage, suggesting similar mechanisms of regulation. Additionally, we have shown that in cartilage the median level of steady-state mRNA for *MMP13 *is approximately 20-fold higher than *MMP28 *and approximately 1,500-fold higher than *ADAMTS16*, with expression of this latter gene approximately 150-fold higher in synovium than cartilage.

This study is the most comprehensive analysis of the metzincin family of proteinases in the joint to date and has identified several proteinase genes not previously reported to be expressed or regulated in synovium.

## Introduction

Osteoarthritis (OA) is a debilitating degenerative joint disease characterized by degradation of articular cartilage. Recent statistics show that approximately 5 million people in the UK suffer from moderate-to-severe OA. These patients are predominantly older than 45 years of age, with the major morbidity in patients over 60 years of age [1]. Given the current demographic trend towards an older population, OA, for which age is an important risk factor, will be an increasing health and economic burden on society.

The molecular mechanisms underlying cartilage destruction in OA are poorly understood (reviewed in [2]). Cartilage is made up of two main extracellular matrix (ECM) macromolecules: type II collagen and aggrecan, a large aggregating proteoglycan [3,4]. The former endows the cartilage with its tensile strength, whereas the latter enables cartilage to resist compression. Quantitatively, more minor components (e.g. type IX, XI and VI collagens, biglycan, decorin and cartilage oligomeric matrix protein) also have important roles in controlling the supramolecular organization of the matrix [3]. Normal cartilage ECM is in a state of dynamic equilibrium, with a balance between synthesis and degradation. For the degradative process, there is a balance between proteinases that degrade the ECM and their inhibitors. In OA, the dogma is that a disruption of this balance, in favour of proteolysis, leads to pathological cartilage destruction.

The matrix metalloproteinases (MMPs) are a family of 23 enzymes in humans that facilitate ECM turnover and breakdown in physiology and pathology [5]. The MMP family contains the only mammalian proteinases that can specifically degrade triple helical collagens at neutral pH. These so-called 'collagenases' specifically cleave a single locus in all three collagen chains at a point three-quarters of the length from the N-terminus of the molecule. The 'classical' collagenases (MMP-1, MMP-8 and MMP-13) have differing substrate specificities for collagens I, II and III, with MMP-13 showing a preference for type II collagen [6]. More recently, gelatinase A (MMP-2) and MT1-MMP (MMP-14) have also been shown to make the specific collagen cleavage, although with less catalytic efficiency than the classical collagenases, at least *in vitro *[7,8]. A second group of proteinases has been identified that affect ECM synthesis and degradation. The 'a disintegrin and metalloproteinase domain with thrombospondin motifs' (ADAMTS) family contains 19 members [9]; these include enzymes involved in collagen biosynthesis as procollagen propeptidases (ADAMTS-2, ADAMTS-3 and ADAMTS-14) [10-13]. Other members of the family are so-called 'aggrecanases' (ADAMTS-1, ADAMTS-4, ADAMTS-5, ADAMTS-8, ADAMTS-9 and ADAMTS-15) that degrade the interglobular domain separating G1 and G2 of aggrecan at a specific Glu373–Ala374 bond [14-18].

A family of four specific inhibitors, the tissue inhibitors of metalloproteinases (TIMPs), has been described [19]. These are endogenous inhibitors of MMPs and potentially the ADAMTS family. The ability of the TIMPs to block active MMPs is largely promiscuous. TIMP-3 seems to be the most potent inhibitor of the ADAMTS family, with a subnanomolar K_i _against ADAMTS-4 and ADAMTS-5 [20].

Although the dogma has been that OA is a 'noninflammatory' disease driven by the chondrocyte, there is certainly evidence for synovial involvement. Low-grade synovitis with thickening of the lining layer, increased vascularity and inflammatory-cell infiltration is reported in patients with all grades of OA [21]. This results in the expression of cytokines and proteinases that could contribute to the pathogenesis of the disease [22]. Arthroscopic studies have also shown that inflammatory changes in the OA synovium often occur where this tissue is in contact with cartilage [23,24]. More recent studies using magnetic resonance imaging (MRI) reinforce the idea that synovitis is a frequent feature, even of early OA [25].

We recently published a complete expression profile of all MMPs, the ADAMTS family and the four TIMPs in normal cartilage compared with OA cartilage [26]. This identified a number of genes that are regulated in OA, which are hence implicated in the disease process. Because the synovium might contribute to cartilage-matrix destruction in OA, we have extended the screening in the current study. Hence, we have profiled *MMP*, *ADAMTS *and *TIMP *genes in both cartilage and synovium from normal and OA joints, giving a more complete picture of proteolysis in this disease.

## Materials and methods

### Cartilage samples

Human articular cartilage was obtained from femoral heads of patients undergoing total-hip-replacement surgery at the Norfolk and Norwich University Hospital (Norwich, UK). Samples from patients with OA (*n *= 16, of which 7 patients were female and 9 patients were male; age range, 61–84 years) were compared with cartilage from patients undergoing hip replacement following fracture to the neck of femur (NOF; *n *= 24, of which 19 patients were female and 5 patients were male; age range, 52–92 years). OA was diagnosed using the clinical history and an examination, coupled with X-ray findings; confirmation of gross pathology was made at time of joint removal. The fracture patients had no known history of joint disease and their cartilage was free of lesions; 80% of these patients underwent surgery within 36 hours of fracture. This study was performed with Ethical Committee approval and all patients provided informed consent.

### Femoral head dissection

Intact femoral heads were washed in sterile PBS. Cartilage samples were removed from the femoral head using a razor blade, chopped into 2–5 mm pieces and snap frozen in liquid nitrogen within 15–30 minutes of surgery. Synovium was washed in sterile PBS, snap frozen and stored in liquid nitrogen.

### RNA extraction from articular cartilage

Extractions were performed on the same day or within 24 hours of dissection from the femoral head following the basic method of Price *et al*. [27]. Cartilage was weighed and ground under liquid nitrogen using a freezer mill (SPEX CertiPrep 6750, Glen Creston, Stanmore, Middlesex, UK). TRIzol^® ^reagent (Invitrogen, Paisley, UK) was added immediately to ground cartilage (1 ml/0.2 g cartilage); the solution was mixed thoroughly and incubated at room temperature for 5 minutes. Ground cartilage was pelleted at 9,500 g for 10 minutes at 4°C and the supernatant recovered. To each 0.5 ml of TRIzol^®^, 300 μl of chloroform was added; the resulting solution was vortexed for 15 seconds and incubated at room temperature 10 minutes. The TRIzol^®^/chloroform solution was centrifuged at 9,500 g for 15 minutes at 4°C. The aqueous layer was recovered into a fresh tube and mixed with a half volume of 100% ethanol. Using the RNeasy Mini Kit (Qiagen, Crawley, West Sussex, UK) samples were applied to spin columns and centrifuged at 9,500 g for 15 seconds at room temperature and the flow-through discarded. Columns were then washed and eluted according to the manufacturer's instructions. RNA samples were quantified using the NanoDrop^® ^spectrophotometer (NanoDrop Technologies, Wilmington, Delaware, USA) and stored at -80°C.

### RNA extraction from synovium

To each 100 mg tissue sample, 1 ml of TRIzol^® ^was added; the solution was homogenized using a TissueLyser (Qiagen, Crawley, West Sussex, UK). The homogenized sample was pelleted at 9,500 g for 10 minutes at 4°C and the supernatant recovered. To each 1 ml of TRIzol^®^, 400 μl of chloroform was added; the resulting solution was vortexed for 15 seconds and incubated at room temperature for 5 minutes. Samples were centrifuged at 9,500 g for 10 minutes at 4°C. The aqueous phase was recovered and 250 μl of isopropanol was added. This was vortexed, as before, and incubated at room temperature for 10 minutes. The mixture was centrifuged at 9,500 g for 10 minutes at 4°C and the supernatant discarded. The RNA pellet was washed with 75% ethanol solution and centrifuged at 9,500 g for 5 minutes at 4°C. The supernatant was discarded, and the pellet was air dried and then suspended in 20 μl of RNAse-free water. The RNA was incubated at 55°C for 10 minutes before storage at -80°C. RNA samples were quantified using the NanoDrop^® ^spectrophotometer (NanoDrop Technologies, Wilmington, Delaware, USA).

### Synthesis of cDNA

cDNA was synthesised from 1 μg of total RNA using Superscript II reverse transcriptase (Invitrogen, Paisley, UK) and random hexamers in a total volume of 20 μl according to the manufacturer's instructions. cDNA was stored at -20°C until used in downstream PCR.

### Quantitative real-time PCR

Oligonucleotide primers and fluorescent-labelled probes were designed using Primer Express 1.0 software (Applied Biosystems, Warrington, UK). Sequences for *MMP *and *TIMP *primers and probes are as described in [28] and sequences for *ADAMTS *primers and probes are as described in [29]. To control against amplification of genomic DNA, primers were placed within different exons close to an intron/exon boundary, with the probe spanning two neighbouring exons where possible. The BLAST was used to search for all the primer and probe sequences to ensure gene specificity. The *18S *rRNA gene was used as an endogenous control to normalize for differences in the amount of total RNA present in each sample; the 18S rRNA primers and probe were purchased from Applied Biosystems (Warrington, UK). Primers for detection of specific splice variants of *MMP28 *were as follows:

1. Variant 1 [GenBank NM_024302]: 5'-CTGCGGCAGTGTCATTGAATG-3' (forward), 5'-GGGCCCCGGAACCT-3' (reverse), and 5'-CATCGACCCCCTTTGAAG-3' (probe).

2. Variant 2 [GenBank NM_032950]: 5'-CTGCGGCAGTGTCATTGAATG-3' (forward), 5'-CCCACGATGGTTGGTATTCATATCA-3' (reverse), and 5'-TTCTTCAAAGTGCAATCCGT-3' (probe).

Relative quantification of genes was performed using the ABI Prism 7700 sequence detection system (Applied Biosystems, Warrington, UK) in accordance with the manufacturer's protocol. PCR reactions contained 5 ng of reverse-transcribed RNA (1 ng for *18S *analyses), 50% TaqMan 2X Master Mix (Applied Biosystems, Warrington, UK), 100 nM of each primer and 200 nM of probe in a total volume of 25 μl. Conditions for the PCR reaction were as follows: 2 minutes at 50°C, 10 minutes at 95°C, 40 cycles of 15 seconds at 95°C, and 1 minute at 60°C.

The threshold cycle (C_T_), the cycle number at which signal is detectable above the baseline, was transformed in two ways. To gain an approximate comparison across all genes measured, amplification efficiency was assumed identical across all primer sets and target gene expression was normalized to *18S *expression using a logarithmic transformation proportional to normalized copy number (log_10_2^-ΔC^T), where ΔC_T _is C_T_(target gene) – C_T_(18S) [30]. When comparing the expression of a single gene across the sample groups, standard curves for each gene were generated using the cDNA from one sample and making twofold serial dilutions across an appropriate range. Relative input amounts of template cDNA were then calculated from C_T _using the standard curves; data are presented as relative levels of mRNA normalized to 18S rRNA. Statistical analyses between OA and fractured NOF samples were performed using this latter method.

For comparison of expression levels between genes, cDNA for *18S*, *MMP13*, *MMP28 *and *ADAMTS16 *was subcloned into a plasmid vector. Serial dilutions of these plasmids, between 10^-12 ^moles and 10^-23 ^moles DNA, were used to create a standard curve. This enabled C_T _to be converted into the number of cDNA molecules and calculation of the ratio of target gene to *18S*.

As a final quality control for the purified RNA samples, only cDNA falling within ± 1.5 C_T _of the median value for *18S *for all samples were used in the downstream study. To ascertain whether the amplification product was indeed that of the desired target gene, products were subcloned and sequenced. All primer and probe sets were shown to amplify specific products from appropriate human tissue samples [28,29].

### Statistical analysis

Differences between control and OA groups were defined using a two-sided Mann-Whitney U test; this nonparametric test makes no prior assumption about the distribution of the data. Correlations between genes were assessed using Spearman's rank correlation.

## Results

### Expression of the *MMP *gene family

**Figure 1:**
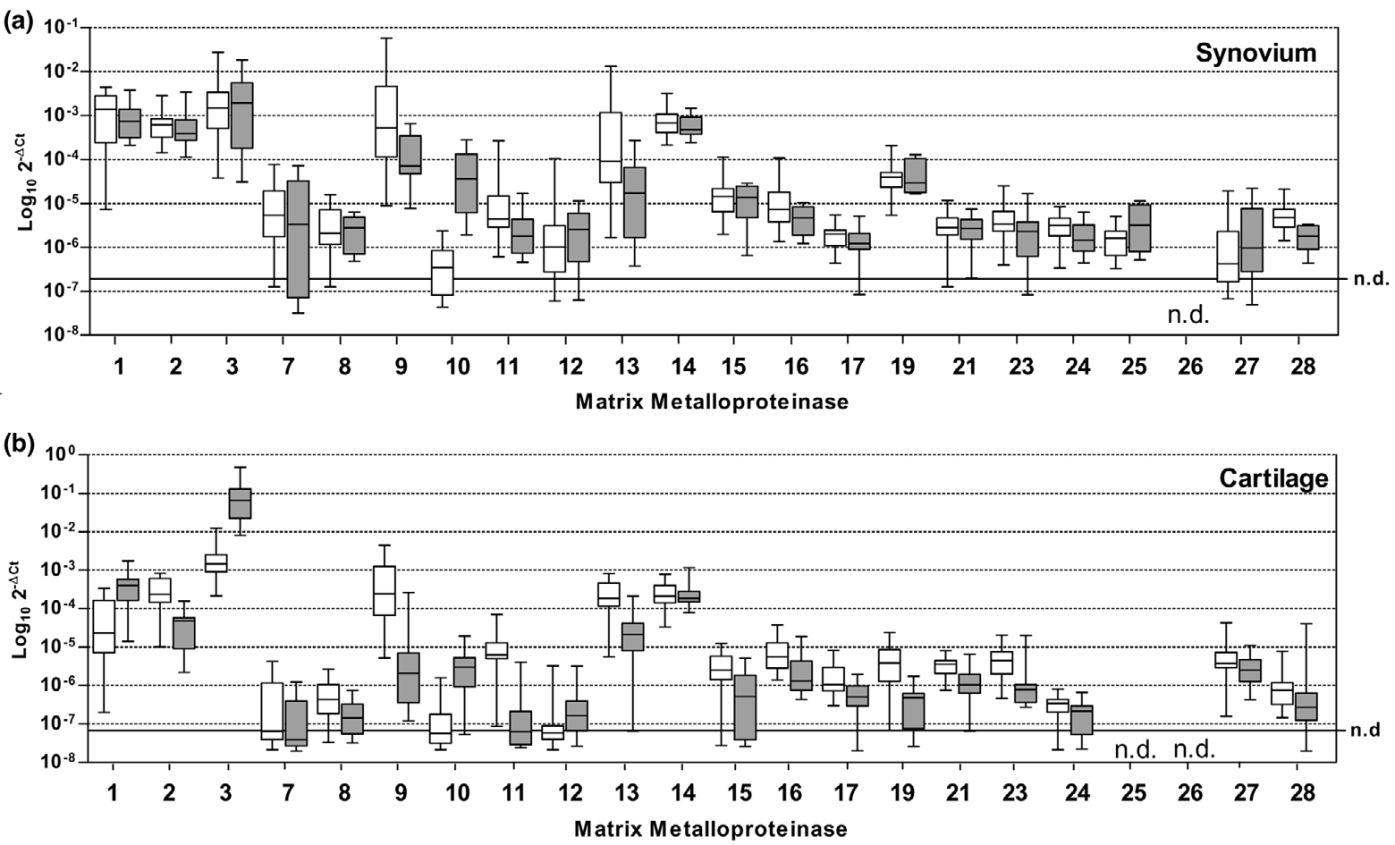
Comparative expression of *MMP *genes in the fractured NOF (filled boxes) compared with OA (open boxes) in hip **(a) **synovium and **(b) **cartilage. The expression level of each gene was determined, as described in Materials and methods, and normalized to the level of *18S *rRNA gene expression using ΔC_T _[C_T_(target gene) – C_T_(18S)]. The box-and-whisker plot shows median values, and each section represents a quartile of the data. Samples on or below the dotted line have C_T_(target gene) = 40 and are therefore not detectable (n.d.).

All *MMP *genes, with the exception of *MMP20 *(which we have previously shown to be expressed neither in cartilage nor in many other tissues), were profiled in synovium and cartilage tissues. OA cartilage was compared with tissue from fractured NOF, with results shown in Figure 1. *MMP9 *(*P *< 0.0001 and *P *= 0.0204), *MMP11 *(*P *< 0.0001 and *P *= 0.0068), *MMP13 *(*P *< 0.0001 and *P *= 0.0037), *MMP16 *(*P *= 0.0014 and *P *= 0.036) and *MMP28 *(*P *= 0.0048 and *P *< 0.0001) were all upregulated in OA, both in cartilage and in synovium (*P *values represent cartilage and synovium, respectively). *MMP10 *(*P *< 0.0001 and *P *< 0.0001) was downregulated in both tissues (*P *values represent cartilage and synovium, respectively). *MMP2 *(*P *< 0.0001), *MMP8 *(*P *= 0.0135), *MMP15 *(*P *= 0.003), *MMP17 *(*P *= 0.0146), *MMP19 *(*P *< 0.0001), *MMP21 *(*P *= 0.0007), *MMP23 *(*P *< 0.0001) and *MMP24 *(*P *= 0.0212) were upregulated in OA cartilage, but no significant change was seen in OA synovium. However, a trend towards an increase of *MMP24 *(*P *= 0.0536) in OA synovium was observed but did not reach statistical significance. *MMP1 *(*P *= 0.0001), *MMP3 *(*P *< 0.0001) and *MMP12 *(*P *= 0.0146) were downregulated in OA cartilage, but no significant change was observed in OA synovium. *MMP25 *and *MMP26 *were not detected in cartilage.

### Expression of the *ADAMTS *gene family

**Figure 2:**
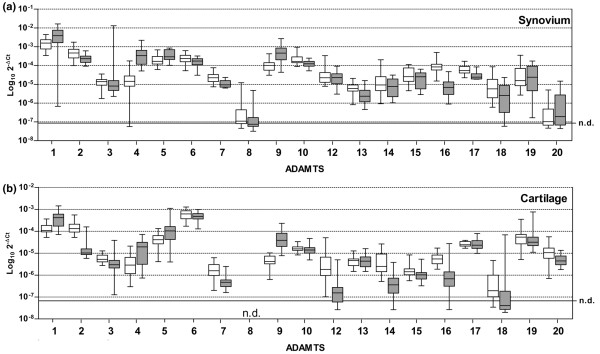
Comparative expression of *ADAMTS *genes in the fractured NOF (filled boxes) compared with OA (open boxes) hip **(a) **synovium and **(b) **cartilage. The expression level of each gene was determined, as described in Materials and methods, and normalized to the level of *18S *rRNA gene expression using ΔC_T _[C_T_(target gene) – C_T_(18S)]. The box-and-whisker plot shows median values, and each section represents a quartile of the data. Samples on or below the dotted line have C_T_(target gene) = 40 and are therefore not detectable (n.d.).

All *ADAMTS *genes were profiled in synovium and cartilage tissues with OA and compared with tissue from fractured NOF (Figure 2). In both cartilage and synovium tissues, *ADAMTS2 *(*P *< 0.0001 and *P *= 0.009), *ADAMTS10 *(*P *= 0.048 and *P *= 0.02) and *ADAMTS16 *(*P *< 0.0001 and *P *< 0.0001) were upregulated, whereas *ADAMTS1 *(*P *= 0.0011 and *P *= 0.0143), *ADAMTS4 *(*P *= 0.0043 and *P *< 0.0001), *ADAMTS5 *(*P *= 0.0135 and *P *= 0.0109) and *ADAMTS9 *(*P *< 0.0001 and *P *< 0.0001) were downregulated compared with the NOF samples. In OA cartilage, *ADAMTS3 *(*P *= 0.0017), *ADAMTS7 *(*P *= 0.0002), *ADAMTS12 *(*P *< 0.0001), *ADAMTS14 *(*P *< 0.0001), *ADAMTS15 *(*P *= 0.045), *ADAMTS18 *(*P *= 0.0033) and *ADAMTS20 *(*P *= 0.0014), were upregulated, but no significant change was seen in synovium. In OA synovium, *ADAMTS8 *(*P *= 0.0017), *ADAMTS13 *(*P *= 0.0045) and *ADAMTS17 *(*P *< 0.0001) were upregulated, but no significant change was seen in cartilage. *ADAMTS8 *was not detected in cartilage tissue and no significant change in expression was seen in *ADAMTS6 *or *ADAMTS19 *genes in either cartilage or synovium.

### Expression of the TIMP gene family

**Figure 3:**
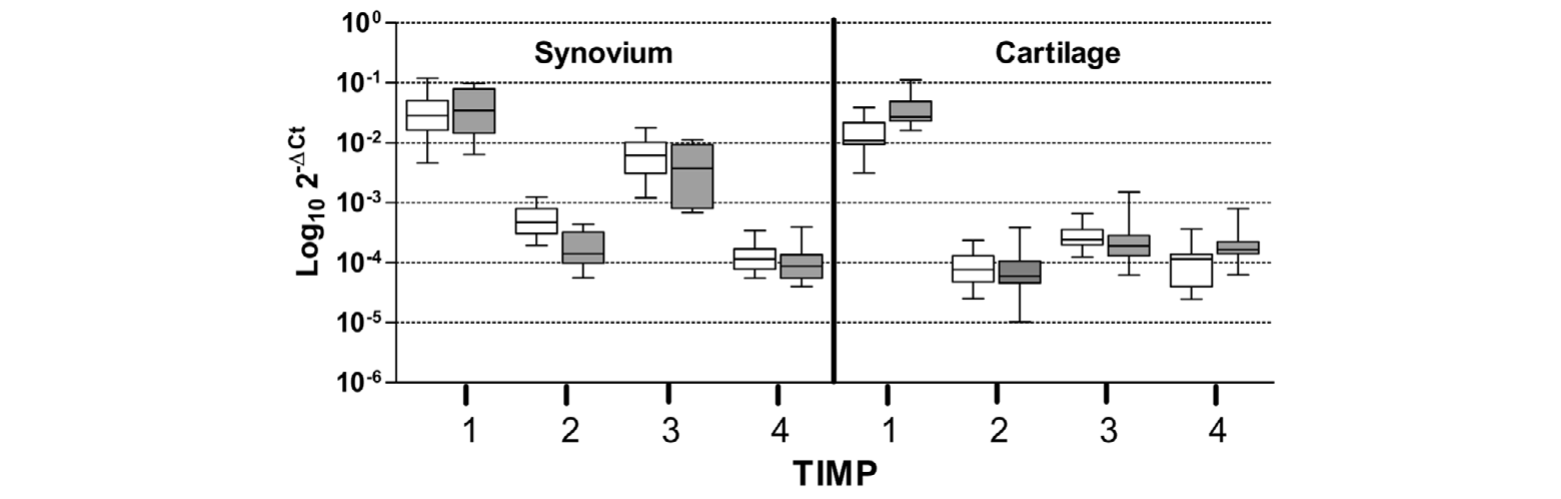
Comparative expression of *TIMP *genes in the fractured NOF (filled boxes) compared with OA (open boxes) hip synovium and cartilage. The expression level of each gene was determined, as described in Materials and methods, and normalized to the level of *18S *rRNA gene expression using ΔC_T _[C_T_(target gene) – C_T_(18S)]. The box-and-whisker plot shows median values, and each section represents a quartile of the data.

All *TIMP *genes were profiled in synovium and cartilage tissues with OA and compared with tissue from fractured NOF (Figure 3). In OA cartilage, *TIMP1 *(*P *< 0.0001) and *TIMP4 *(*P *= 0.0033) were downregulated. No change was seen in *TIMP2 *or *TIMP3 *levels. In OA synovium, *TIMP2 *(*P *< 0.0001) was upregulated, but no significant changes were seen in *TIMP1*, *TIMP3 *or *TIMP4 *levels.

### Correlation of gene expression between tissues

**Figure 4:**
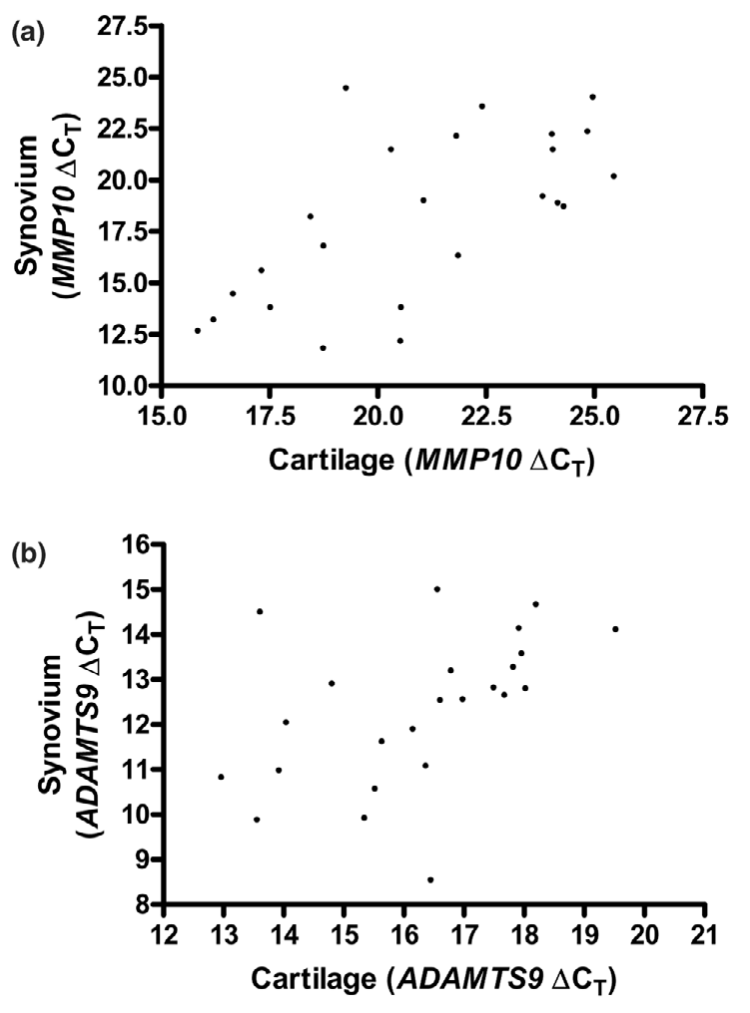
Correlation analysis of gene expression in cartilage compared with synovium. Correlation plots for **(a) ***MMP10 *and **(b) ***ADAMTS9 *expression in cartilage and synovium taken from the same joint. Correlation coefficients are as follows: *MMP10*, r = 0.645 (*P *= 0.0006); and *ADAMTS9*, r = 0.590 (*P *= 0.0024).

For 11 OA and 13 NOF samples, cartilage and synovium taken from the same joint was analysed. Although these sample numbers are small for correlation analysis, a number of genes show a correlation in expression between tissues: *MMP9 *(r = 0.584; *P *< 0.01), *MMP10 *(r = 0.645; *P *< 0.001), *MMP12 *(r = -0.439; *P *< 0.05), *MMP17 *(r = 0.418; *P *< 0.05), *MMP23 *(r = 0.411; *P *< 0.05), *MMP28 *(r = 0.412; *P *< 0.05), *ADAMTS4 *(r = 0.521; *P *< 0.01), *ADAMTS9 *(r = 0.611; *P *< 0.002), and *ADAMTS16 *(r = 0.398; *P *= 0.054). Figure 4 shows the two most significant correlations.

### Comparison of *MMP13*, *MMP28 *and *ADAMTS16 *expression

**Figure 5:**
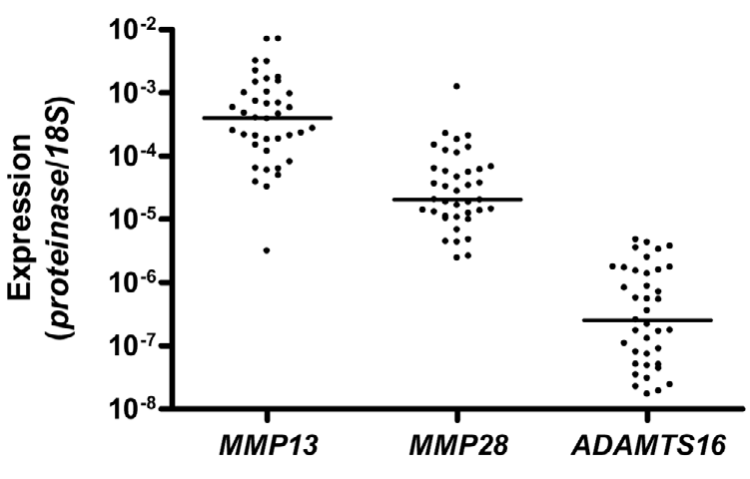
Comparison of *MMP13*, *MMP28 *and *ADAMTS16 *expression levels in cartilage. Expression of each gene was quantified against standard curves constructed using purified cDNA and normalized to *18S *expression, which was similarly quantified. Median values are marked.

In our previous work [26], the identification of *MMP28 *and *ADAMTS16 *as genes significantly upregulated in OA cartilage raised the question of how their expression levels compared with that of *MMP13*, the MMP pathognomic of collagen destruction in OA. Hence, we subcloned cDNA for each gene and used these to establish a standard curve of the absolute number of molecules of cDNA against the C_T _value. Figure 5 shows that the median level of expression of *MMP13 *is approximately 20-fold higher than that of *MMP28 *in cartilage and approximately 1,500-fold higher than *ADAMTS16*. The median level of *MMP13 *is similar in cartilage and synovium, whereas *MMP28 *is expressed at an approximately eightfold higher level and *ADAMTS16 *is expressed at an approximately 150-fold higher level in synovium compared with cartilage (data not shown).

### Expression of *MMP28 *splice variants

Two splice variants of *MMP28 *are listed on the database [GenBank NM_024302 and NM_032950]. The longer mRNA includes exon 8, which contains an in-frame stop codon, leading to a protein truncated in the C-terminal hemopexin-like domain; the shorter mRNA excludes exon 8 and encodes a protein with a complete C-terminal domain. Taqman primer/probe sets were designed to distinguish between these variants and verify them against cloned cDNA. This demonstrated that only the shorter mRNA (encoding the full-length protein) was expressed at a detectable level in cartilage (data not shown).

## Discussion

This is the first study to profile the expression of all *MMP*, *ADAMTS *and *TIMP *genes in synovium and compare tissue from OA patients with those who have a fracture to the NOF. Konttinen *et al*. had previously used conventional reverse-transcriptase PCR to profile the expression of 16 *MMPs *in synovium (*MMP1–3*, *MMP7-17 *and *MMP19*), with induction of *MMP1*, *MMP9*, *MMP13*, *MMP14 *and *MMP15 *in synovium from rheumatoid arthritis compared with trauma controls [31].

Recent studies show that inflammatory changes in the OA synovium are variable throughout the tissue [23,24]. This might be expected to confound measurement of regulated gene expression in this tissue because variation across the tissue within a single joint would lead to greater variation in gene expression within each patient group. This is borne out by our study, in which more genes showed significant regulation between OA and fractured NOF in cartilage than synovium.

Several studies have measured MMPs in synovium or synovial fluid and demonstrated that levels of MMP-1 and MMP-3 are elevated in rheumatoid arthritis (compared with OA or other control groups, such as joint trauma; e.g. [32-36]). High expression of a number of MMPs has been demonstrated in rheumatoid pannus, suggesting a role mediating cartilage destruction in RA (e.g. [31,37]). Indeed, levels of synovial fluid MMP-1 and MMP-3 correlate to synovial hyperplasia [38]. Although levels of synovial fluid MMPs are generally raised in RA compared with OA, the levels of at least MMP-1–3, MMP-8, MMP-9 and TIMP-1 are reported to be raised in OA compared with control, nonarthritic, synovial fluid (e.g. [39,40]).

In this study, the four most significantly upregulated genes (*P *< 0.0001) in OA synovium compared with fracture of the NOF are *MMP28*, *ADAMTS16*, *ADAMTS17 *and *TIMP2*. Interestingly, we recently published the first report of *MMP28 *and *ADAMTS16 *gene expression in cartilage and their upregulation in OA [26]. Although these enzymes have no known function, the fact that their expression is switched on in at least two tissues of the OA joint underlines their probable role in this disease. *ADAMTS17 *is another recently cloned ADAMTS family member with unknown function and this is the first report of its expression in synovium. TIMP-2 can inhibit the activity of all the MMPs and the increase in expression in OA synovium might represent an attempt to control proteolysis. The expression of *MMP13*, the probable key collagenase in OA, is also upregulated in OA synovium, in addition to cartilage.

MMP-1, MMP-2, MMP-9–11, MMP-13 and TIMP-2 are all expressed in human growth plate hypertrophic chondrocytes [41]. Interestingly, a significant subset of these (*MMP2*, *MMP9*, *MMP11*, *MMP13 *and *TIMP2*) is upregulated in OA cartilage.

MMP-1 and MMP-3 are the most frequently measured enzymes in RA, where correlations of synovial fluid or serum levels to disease activity have been reported [36,39]. Our data show that in synovium there is no difference in expression between OA and fracture to the NOF, whereas in cartilage, as we and others have previously reported, there is a significant downregulation of expression in OA [26,42]. This might reflect the late stage of the disease tissue in our study (see below), or, for MMP-3, a maintenance role in cartilage metabolism. Our data support the fact that *MMP3 *is a highly expressed gene in synovium, as previously reported [37].

Other genes expressed at significantly higher levels in OA synovium compared with fracture to the NOF are *MMP9*, *MMP11*, *MMP16*, *ADAMTS8*, *ADAMTS10 *and *ADAMTS13*, although the absolute expression of *ADAMTS8 *is low. MMP-9 has previously been reported as elevated in RA synovium (e.g. [31,38]) and in synovium and synovial fluid in a dog model of OA [43]. Elevated *MMP11 *expression has also been previously reported in late-stage OA [42]. *MMP16 *expression has been reported in synovium, but was not associated with disease [31], whereas *ADAMTS8*, *ADAMTS10 *and *ADAMTS13 *have not previously been described in synovium. ADAMTS-8 is capable of degrading aggrecan [44], although with lesser catalytic efficiency compared with, for example, ADAMTS-4 or ADAMTS-5.

The expression of *MMP10 *was the most significantly downregulated of any *MMP *in OA synovium compared with fractured NOF. *MMP10 *expression has been previously described in synovium, but its regulation has not been described in disease [31]. *MMP10 *expression has also been associated with the invasive potential of synovial fibroblasts in both OA and RA [45].

We previously reported that the expression of *ADAMTS4*, *ADAMTS5 *and *ADAMTS9*, all aggrecanases, was downregulated in OA cartilage compared with fractured NOF [26]. These genes, particularly *ADAMTS4 *and *ADAMTS9*, are also downregulated in OA synovium. This could reflect a role for aggrecanases in the earlier phases of OA, but similar expression patterns in cartilage and synovium could also suggest a role for the synovium in aggrecan turnover and breakdown. Synovium has been shown to express soluble aggrecanase activity [46], and ADAMTS-5 has been localized to human synovium [47].

A comparison of the expression patterns of *MMP*, *ADAMTS *and *TIMP *genes in cartilage in this study with those in our previous report shows predominantly similar data. Overall, a greater number of genes were regulated between OA and fractured NOF samples in the current study. This might reflect differences in the actual patient groups in each study or the rapidity of tissue dissection and processing. Genes induced in OA with weak significance in the current study, *MMP17*, *MMP24 *and *ADAMTS10*, all show a trend towards induction in our previous cohort but did not reach significance. The same is true for *MMP12*, *MMP15 *and *ADAMTS3*. *ADAMTS15 *showed upregulation in OA in the current study at *P *< 0.05 and similarly significant downregulation in our previous study. This might represent variation because of multiple testing. More difficult to explain are the significantly upregulated genes in OA in the current study, *MMP21 *(*P *< 0.001), *MMP19 *(*P *< 0.0001) and *MMP23 *(*P *< 0.0001); these genes showed no significant difference in our earlier cohort. Where genes are annotated as 'not detected' (Figures 1, 2, 3 and [26], these might not be expressed at all in the tissue or the median C_T _is 40, with a few samples showing a low level of expression.

For *MMP9*, *MMP10*, *MMP12*, *MMP17, MMP23*, *MMP28*, *ADAMTS4*, and *ADAMTS9*, there is a significant correlation between expression levels in the synovium and cartilage. This presumably reflects the fact that these tissues are exposed to a similar environment in terms of growth factors and cytokines. For genes where expression between the two tissues does not correlate, there must therefore be other factors driving expression, such as mechanical load, oxidative stress and cell-matrix interaction.

To resolve previously raised questions, we used plasmid cDNA for *MMP13*, *MMP28 *and *ADAMTS16 *to construct standard curves for these genes. This demonstrated that *MMP13 *was the most abundant mRNA of the three genes. It remains impossible to speculate about their relative importance because the substrates, and therefore the specific activities of MMP-28 and ADAMTS-16, are unknown.

## Conclusion

This study is the most comprehensive analysis of the metzincin family of proteinases in the joint to date and has identified several proteinase genes not previously reported to be expressed or regulated in synovium. Future work will focus on the function of key genes. The data also show coordinate regulation of a subset of genes throughout the synovium and cartilage that might inform analyses of regulatory pathways and transcription factors.

## Abbreviations

ADAMTS = a disintegrin and metalloproteinase domain with thrombospondin motifs; BLAST = basic local alignment search tool; C_T _= threshold cycle; ECM = extracellular matrix; K_i _= inhibition constant; MMP = matrix metalloproteinase; MRI = magnetic resonance imaging; NOF = neck of femur; OA = osteoarthritis; PBS = phosphate-buffered saline; PCR = polymerase chain reaction; RA = rheumatoid arthritis; TIMP = tissue inhibitor of metalloproteinases.

## Competing interests

The authors declare that they have no competing interests.

## Authors' contributions

RKD helped design and coordinate the study, collected and processed tissue samples, performed real-time PCR, analysed data and helped draft the manuscript; JGW and LK helped in collecting and processing tissue samples; CD and AC took patient consent and coordinated tissue collection; STD helped design and coordinate study, tissue collection and interpretation of data; IMC helped conceive, design and coordinate the study, and helped to draft the manuscript. All authors read and approved the final manuscript.

## Figures and Tables

**Table 1:** Genes with changing expression levels in cartilage or synovium when comparing OA with fracture to the NOF

***P *value**	**Cartilage**		**Synovium**	
	**Up**	**Down**	**Up**	**Down**

**<0.05**	*MMP8*	*ADAMTS5*	*MMP9*	*ADAMTS1*
	*MMP17*		*MMP16*	*ADAMTS5*
	*MMP24*		*ADAMTS10*	
	*ADAMTS10*			
	*ADAMTS15*			
**<0.01**	*MMP15*	*MMP12*	*MMP11*	
	*MMP16*	*ADAMTS1*	*MMP13*	
	*MMP28*	*ADAMTS4*	*ADAMTS2*	
	*ADAMTS3*	*TIMP4*	*ADAMTS8*	
	*ADAMTS18*		*ADAMTS13*	
	*ADAMTS20*			
**<0.001**	*MMP21*			
	*ADAMTS7*			
**<0.0001**	*MMP2*	*MMP1*	*MMP28*	*MMP10*
	*MMP9*	*MMP3*	*ADAMTS16*	*ADAMTS4*
	*MMP11*	*MMP10*	*ADAMTS17*	*ADAMTS9*
	*MMP13*	*ADAMTS9*	*TIMP2*	
	*MMP19*	*TIMP1*		
	*MMP23*			
	*ADAMTS2*			
	*ADAMTS12*			
	*ADAMTS14*			
	*ADAMTS16*			
